# Three-Dimensional Model of Sub-Plasmalemmal Ca^2+^ Microdomains Evoked by the Interplay Between ORAI1 and InsP_3_ Receptors

**DOI:** 10.3389/fimmu.2021.659790

**Published:** 2021-04-28

**Authors:** Diana Gil, Andreas H. Guse, Geneviève Dupont

**Affiliations:** ^1^ The Ca^2+^ Signalling Group, Department of Biochemistry and Molecular Cell Biology, University Medical Center Hamburg-Eppendorf, Hamburg, Germany; ^2^ Unit of Theoretical Chronobiology, Faculté des Sciences CP231, Université Libre de Bruxelles (ULB), Brussels, Belgium

**Keywords:** non-TCR/CD3-dependent microdomains, Calcium Signaling, T-cells, COMSOL, computational model, Store operated calcium entry (SOCE)

## Abstract

Ca^2+^ signaling plays an essential role in T cell activation, which is a key step to start an adaptive immune response. During the transition from a quiescent to a fully activated state, Ca^2+^ microdomains characterized by reduced spatial and temporal extents are observed in the junctions between the plasma membrane (PM) and the endoplasmic reticulum (ER). Such Ca^2+^ responses can also occur in response to T cell adhesion to other cells or extracellular matrix proteins in otherwise unstimulated T cells. These non-TCR/CD3-dependent Ca^2+^ microdomains rely on d-*myo*-inositol 1,4,5-trisphosphate (IP_3_) signaling and subsequent store operated Ca^2+^ entry (SOCE) *via* the ORAI/STIM system. The detailed molecular mechanism of adhesion-dependent Ca^2+^ microdomain formation remains to be fully elucidated. We used mathematical modeling to investigate the spatiotemporal characteristics of T cell Ca^2+^ microdomains and their molecular regulators. We developed a reaction-diffusion model using COMSOL Multiphysics to describe the evolution of cytosolic and ER Ca^2+^ concentrations in a three-dimensional ER-PM junction. Equations are based on a previously proposed realistic description of the junction, which is extended to take into account IP_3_ receptors (IP_3_R) that are located next to the junction. The first model only considered the ORAI channels and the SERCA pumps. Taking into account the existence of preformed clusters of ORAI1 and STIM2, ORAI1 slightly opens in conditions of a full ER. These simulated Ca^2+^ microdomains are too small as compared to those observed in unstimulated T cells. When considering the opening of the IP_3_Rs located near the junction, the local depletion of ER Ca^2+^ allows for larger Ca^2+^ fluxes through the ORAI1 channels and hence larger local Ca^2+^ concentrations. Computational results moreover show that Ca^2+^ diffusion in the ER has a major impact on the Ca^2+^ changes in the junction, by affecting the local Ca^2+^ gradients in the sub-PM ER. Besides pointing out the likely involvement of the spontaneous openings of IP_3_Rs in the activation of SOCE in conditions of T cell adhesion prior to full activation, the model provides a tool to investigate how Ca^2+^ microdomains extent and interact in response to T cell receptor activation.

## Introduction

T cell stimulation initiates a cascade of intracellular events among which increases of the free cytosolic Ca^2+^ concentration (C_C_), having well-defined spatio-temporal characteristics, play a crucial role. In particular, Ca^2+^ controls many processes that are essential for an adaptive immune response such as transcriptional activation, proliferation, differentiation or secretion of cytokines (1). As in most non-excitable cells, the classical model for Ca^2+^ signaling in T cells considers as first step the formation of d-*myo*-inositol 1,4,5-trisphopshate (IP_3_) and subsequent activation of IP_3_R (2). Another Ca^2+^ mobilizing second messenger, nicotinic acid adenine dinucleotide phosphate (NAADP) has been shown to be also involved through its action on type 1 ryanodine receptor (RYR1) (3, 4). Both IP_3_R and RyR1 release Ca^2+^ from the endoplasmic reticulum (ER), which results in a significant decrease of the luminal Ca^2+^ concentration and thereby stimulates capacitative or store operated Ca^2+^ entry (SOCE) (see (5) for review) *via* the ORAI/STIM system. The Ca^2+^ filling state of the ER is indeed detected by Ca^2+^ sensors stromal interaction molecules 1 (STIM1) and 2 (STIM2) located in the ER membrane (6, 7). Upon a decrease in ER Ca^2+^, Ca^2+^ dissociates from STIMs and STIM molecules aggregate and move to so-called “junctional spaces”. These junctions, also called “puncta”, are regions of the cell where the ER is located in close proximity of the plasma membrane (PM), thereby creating a 10-20 nm gap of cytosol between the two membranes (8). There, STIM molecules can recruit ORAI1 to form active Ca^2+^ channels that allow Ca^2+^ to enter from the extracellular medium into the cytosol (1, 9). The relation between SOCE and the ER Ca^2+^ concentration (C_S_) is non-linear and generally described by a decreasing Hill function of C_S_, with a K_D_ for half activation of the order of ~200 µM when it depends on the dissociation of Ca^2+^ from STIM1 and of ~400 µM when it depends on the dissociation of Ca^2+^ from STIM2 (6, 10, 11). Since resting C_S_ is in the range 400-600 µM depending on the cell type (12), STIM1 is activated only upon conditions of massive ER depletion, while STIM2 has a greater sensitivity to small decreases in C_S_.

Global Ca^2+^ signaling is often preceded by local Ca^2+^ signals known as “Ca^2+^ microdomains” that occur as a signaling transition state between quiescence and full activation of cells (13). Interestingly, a role for ORAI/STIM proteins in Ca^2+^ microdomains occurring in the process of immune synapse formation was described (14). High-resolution Ca^2+^ imaging revealed that T cell Ca^2+^ microdomains occur as a signaling transition during the first ~15 s following cell activation by TCR/CD3 and show well-defined spatio-temporal dynamics. Such Ca^2+^ increases appear randomly in time and last for 64 ± 3 ms [computed from the data of (4)]. Ca^2+^ microdomains extent on 0.216 μm^2^ ± 0.004 and display an average amplitude of 325 ± 11 nM (3, 4). They are initiated by NAADP signaling acting on RYR1 and strongly depend on ORAI1 and both STIM1 and STIM2.

Similar Ca^2+^ microdomains can moreover occur in the absence of T cell activation *via* TCR/CD3 (non-TCR/CD3-dependent Ca^2+^ microdomains). These small signals are more than four times less frequent than those occurring in the first seconds following stimulation and are shorter [44 ± 4 ms, computed from the data of (4)] but display similar amplitudes (290 ± 12 nM). This amplitude is not affected by the absence RYR1, which indicates that NAADP-evoked Ca^2+^ release is not significantly involved in the creation of non-TCR/CD3-dependent Ca^2+^ microdomains. In contrast to TCR/CD3-dependent Ca^2+^ microdomains, the non-TCR/CD3-dependent microdomains rely on T cell adhesion to other cells or proteins of the extracellular matrix (ECM), integrin evoked IP_3_ signaling and subsequent SOCE *via* the ORAI/STIM system (15). In these conditions, pre-formed complexes of ORAI1 and both STIM1 and STIM2 have been obtained by FRET experiments and super-resolution microscopy (4, 15).

Non-TCR/CD3-dependent Ca^2+^ microdomains are the signature of a basal, low-level Ca^2+^ entry in resting T cells. How partial and local depletion in C_S_ allows Ca^2+^ to enter into the cytosol through preformed assembly of ORAI1 and STIM molecules, cannot be resolved experimentally on the single channel level given that current Ca^2+^ imaging systems are characterized by spatial and temporal resolutions in the range of several hundreds of nanometers and tens of milliseconds, e.g. 368 nm and 20 to 25 ms, respectively (3). Here, we used mathematical modelling to further investigate the molecular origin of non-TCR/CD3-dependent Ca^2+^ microdomains. In particular, we quantitatively explored the relation between the Ca^2+^ concentration in a PM junction and in its adjacent ER upon opening of pre-formed ORAI1/STIM2 channels, under different conditions triggering channel opening and for various spatial geometries of the junction.

Mathematical modeling has been much used in the field of Ca^2+^ signaling to help deciphering its sophisticated spatio-temporal organization and its versatility (16). In particular, small-scale Ca^2+^ events corresponding to the spontaneous, stochastic opening of a few IP_3_R have been much investigated theoretically (17–22). Similarly, simulations of Ca^2+^ fluxes in realistic geometries have been very useful to explore the relation between microscopic structures and cellular responses in cardiac cells (23, 24).

How SOCE interferes with Ca^2+^ signaling has mainly been investigated in models that do not take the detailed arrangement of the Ca^2+^ stores and fluxes into account. Using an *ad hoc* dependence of the SOC current on ER Ca^2+^ concentration, Ong et al. (25) showed that the re-localization of STIM1 near ORAI1 channels, and thus the status of SOCE, depends on the Ca^2+^ concentration just beneath the ER membrane. A molecular description of a Ca^2+^-dependent ORAI1-STIM1 binding was introduced by Liu et al. (26), who fitted their model to reproduce data of SOCE activation in human Jurkat leukemic T cells (10). A theoretical study closely based on experimental observations in airway smooth muscle cells enlightened the primary role played by SOCE in the maintenance of agonist induced Ca^2+^ entry (27). A similar, phenomenological description of SOCE was introduced in an extended model of Ca^2+^ signaling and transcription factor activation in T-lymphocytes (28). In another recent study, Yoast et al. (29) used mathematical modelling to back-up their experimental investigation of the relation between the precise composition of ORAI heteromultimers and global Ca^2+^ signaling that differentially activates NFAT-mediated transcriptional responses.

Other mathematical models have focused on the spatial aspects of SOCE-mediated influx, and more especially on the influence of the geometry of the ER-PM junction on the spatio-temporal characteristics of Ca^2+^ entry. These models describe Ca^2+^ signaling in one PM-ER junction. Unequivocal theoretical considerations allowed establishing the highly local character of the ORAI1-mediated Ca^2+^ profile through ORAI1 in a realistic junction geometry (30). A 3D spatial model was developed by Samanta et al. (31) to investigate the effect of clustering within the ER-PM junction of RBL-1 cells. They concluded that ORAI1 clustering increases both the amplitude and the spatial spread of the Ca^2+^ signal in the junction in response to a massive decrease in ER Ca^2+^. A more detailed description of the junction, taking into account the sub-PM ER and the activity of the SERCAs in the junction, was later developed by McIvor et al. (32). The model allowed to investigate the microscopic patterning of the Ca^2+^ signal in the junction and the replenishment of the ER after significant depletion, in conditions encountered when cells are treated with massive doses of SERCA inhibitors.

In this study, we adapt the model proposed by McIvor et al. (32) to investigate the molecular origin of the non-TCR/CD3-dependent Ca^2+^ microdomains that have been observed in T cells adhering to poly-L-lysine coated surface (4). After describing the mathematical model, we successively simulate situations with and without IP_3_Rs in the sub-PM ER membrane. We found that the local ER depletions created by their spontaneous activity are necessary to account for experimental observations about the average amplitude of Ca^2+^ microdomains in T cells. We also used the model to investigate the impact of Ca^2+^ diffusion in the ER and of the distance between ORAI1 channels and found that only the former factor has a significant impact on the characteristics of the Ca^2+^ signals in the PM-ER junction.

## Description of the Mathematical Model

Our model is based on the realistic 3-dimensional mathematical description of the junction proposed by McIvor et al. (32). In this study, the authors investigated Ca^2+^ dynamics associated with SOCE in conditions corresponding to a significant depletion of the ER, up to [Ca^2+^] = 150 μM. Here, we used a modified version of this model to investigate the origin of the non-TCR/CD3-dependent Ca^2+^ microdomains described by Diercks et al. (4).

### Spatial Geometry

**Figure 1:**
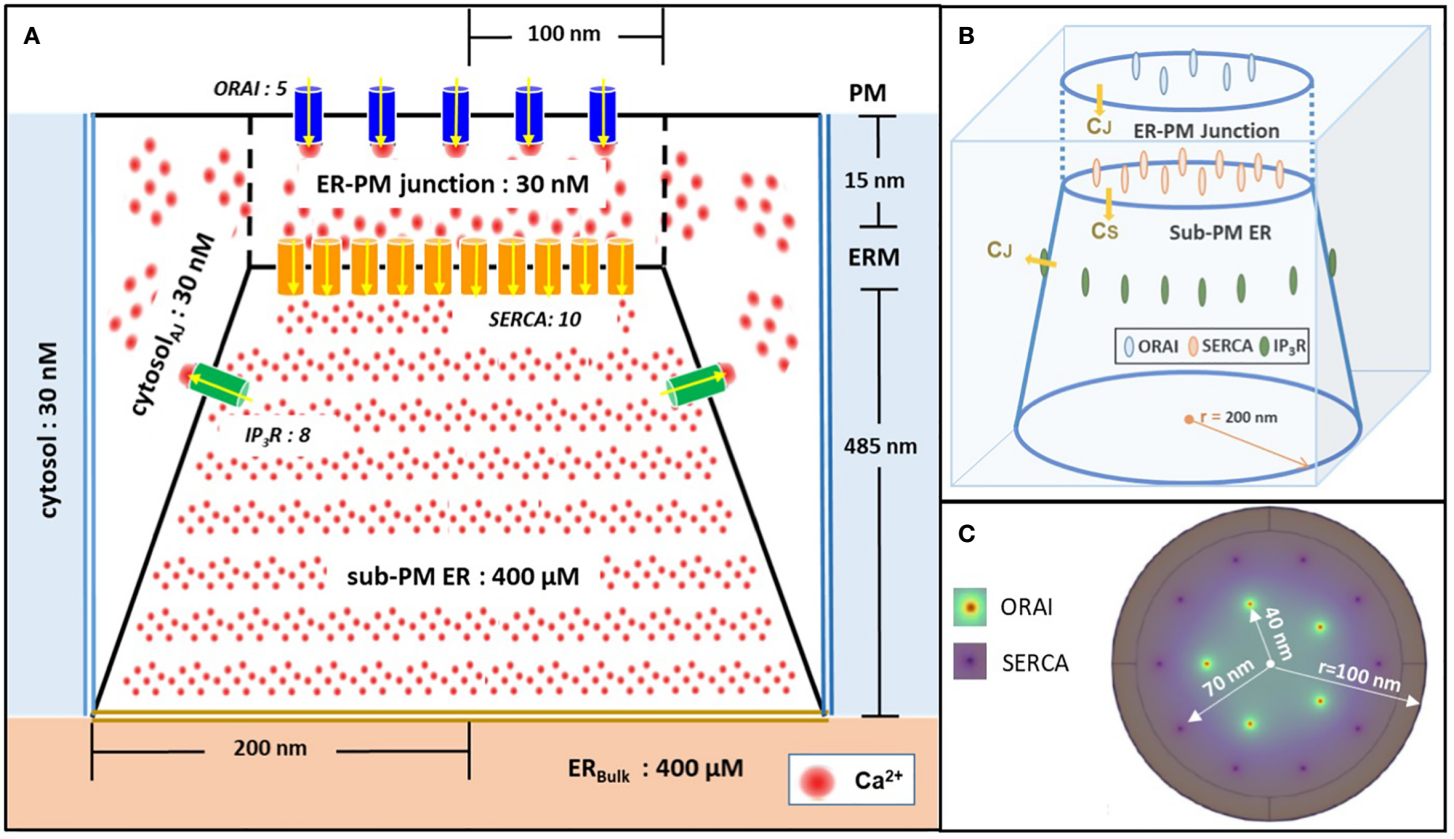
Schematic representation of the model geometry of the ER-PM junction and sub-PM ER used to investigate the origin of the Ca^2+^ microdomains in T cells. **(A)** Frontal diagram showing the dimensions of the cone that represents the sub-PM ER, of the junction and of the portion of the cytosol considered in the simulations. ORAI1 channels are in blue, SERCA pumps in yellow and IP_3_Rs in green. Plain lines represent membrane boundaries; dashed lines, fictitious limits between the junction and the cytosol and double lines indicate the limits of the simulated system. The resting Ca^2+^ concentrations considered as initial conditions and boundary conditions in the two compartments are indicated. **(B)** 3D view of the model geometry **(C)** Upper view of the positions of the ORAI1 channels on the PM, in green, and of the ERM, in purple, using COMSOL. Not to scale. This geometry is based on McIvor et al. (32). See text for details.

Model geometry is shown in **Figure 1**. It represents a portion of ER surrounded by cytoplasm, with the specificity that the ER and the PM membranes are very close to one another. The part of the ER that is closest to the PM is called sub-PM ER (25) and is described by a conic domain. The up and bottom faces have diameters of 200 nm and 400 nm, respectively. The depth of the sub-PM is 485 nm (32). The bottom face stands for the sub-PM ER limit, where it coincides with the bulk of the ER (ER_Bulk_). The sub-PM ER is embedded in a cubic cytosolic domain of 400 nm x 400 nm that includes the ER-PM junction itself, represented as a cylindrical sub-domain of 200 nm in diameter that extends 15 nm from the ER membrane (ERM) up to the PM. The rest of the cube corresponds to the cytosol adjacent to the junction (**Figures 1A, B**). The lateral faces of the cube are in contact with the bulk cytosol of the cell.

We include five ORAI channels on the PM as estimated by Hogan (30) and ten SERCA pumps as proposed by McIvor et al. (32), both placed at the rim of concentric rings with a 40 nm and 70 nm radius, respectively (**Figure 1C**). The corresponding inter-channel distances are thus 47 nm for ORAI1s, as estimated by Samanta et al. (31) and 44 nm for SERCAs. The arrangement of SERCAs as a ring around the ORAI1s in the junction follows the arrangement proposed by Alonso et al. (33). As will be explained in the “Results” section (15), IP_3_Rs have to be considered in the simulations to describe junctional Ca^2+^ microdomains in T cells. Thus, a cluster of eight IP_3_Rs is included at the boundary between the sub-PM ER and the cytosol. Spatial arrangement of the IP_3_Rs is based on the work by Thillaiappan et al. (34), who reported clusters of native immobile ‘licensed’ IP_3_Rs residing alongside the ER-PM junction and facing the PM. The IP_3_R inter-channel distance varies from 30 nm up to 200 nm (34–36). We chose 90 nm for symmetry reasons and use the same distance to the ER membrane. Actually, as detailed in the next section, this location simply reflects that IP_3_R’s colocalize with SOC channels.

Continuous black lines in **Figure 1A** represent membranes and thus correspond to no flux boundary conditions, except across channels and pumps. The black dashed lines represent symbolic boundaries between the ER-PM junction and the cytosol, through which Ca^2+^ actually diffuses freely. Dirichlet boundary conditions corresponding to the initial Ca^2+^ concentrations are imposed at the outer boundaries represented by double bold lines, blue to the cytosol and yellow to ER_bulk_ as in McIvor et al. (32). Thus, we fix the free cytosolic [Ca^2+^] C_C_ at 30 nM at the boundaries between the junction and the cytosol. This is in agreement with the brief and local nature of Ca^2+^ microdomains (30). Similarly, because the regulation of SOCE is governed mainly by [Ca^2+^]_ER_ in close proximity to the junction (25), a constant ER Ca^2+^ concentration equal to 400 μM, corresponding to the Ca^2+^ resting concentration in the bulk of the ER, is imposed at the bottom boundary of the sub-PM ER domain. All parameter values are given in **Table S1**.

### Ca^2+^ Dynamics

The model describes the evolutions of Ca^2+^ concentration in the cytosol (*C_C_*) and in the sub-PM ER (*C_s_*). These concentrations obey the diffusion equations

(1a) $$\frac{\partial C_{C}}{\partial t}-D_{C}\nabla^{2}C_{C}=0\;,$$

(1b) $$\frac{\partial C_{S}}{\partial t}-D_{S}\nabla^{2}C_{S}=0\;,$$

where D_C_ and D_S_ stand for the Ca^2+^ diffusion coefficient in the cytosol and in the ER, respectively. Default values for these coefficients are 220 and 10 μm^2^/s (32). Ca^2+^ fluxes are taken into account in the boundary conditions that are schematized in **Figure 1**, described in the previous section and detailed in the **Supplementary Information**.

The flux through the ORAI channel is given by Eq. 2, with *I_ORAI_* the maximal single channel current, *F* the Faraday constant, *z* the charge of a Ca^2+^ ion and *A_O_* the surface of the channel pore:

(2) $$J_{ORAI}=f\left(C_{S}^{loc}\right)\frac{I_{ORAI}}{F\cdot z\cdot A_{o}}$$

The function *f*, which takes discrete values between 0 and 1, allows to consider the different opening states of the ORAI1 channel. These states relate to the local concentration of Ca^2+^ around the mouth of the IP_3_R $(C_{S}^{loc}$)

In the model, this concentration was evaluated as the average [Ca^2+^] in a 108 nm^3^ volume surrounding each IP_3_R. Each volume is independently sensed by one associated ORAI1 channel. This modelling assumption, through which we explicitly consider SOC channels located in very close proximity of immobile IP_3_Rs, is based on considerations described in Thillaiappan et al. (34). Eq. 2 implies that the exact distance between ORAI1’s and IP_3_R’s will not change the outcome of the simulations, as a consequence of the fact that STIM molecules are not modelled explicitly. Function *f* was defined on the basis of the relation between the SOC current and [Ca^2+^]_ER_ measured by Luik et al. (10) in Jurka T cells, while taking into account that STIM2 has a lower K_D_ for Ca^2+^ than STIM1. This relation was combined with the results of Li et al. (37) on the graded activation of ORAI1 channels in HEK cells (see **Figure S1** and **Supplementary Information** for a detailed explanation). A similar graded description of the different opening states of ORAI1 depending in that case on the number of STIM1 attached is used in Schmidt et al. (38). Because we investigate conditions of full ER, STIM1 that has a high affinity for Ca^2+^ is not explicitly considered in the present simulations (39).

The flux through the IP_3_R is given by Eq. 3, with *I*_IP3_ *_R_* the current through the IP_3_R and *A*_IP3_ *_R_* the surface of the channel pore. The second factor allows to scale the current to take the actual gradient across the channel pore into account, where *C_S,0_* and *C_J,0_* represent resting concentrations of Ca^2+^ in the ER and in the cytosol (40). A unitary IP_3_R current of 0.064 pA (41) has been estimated at the baseline concentration difference.

(3) $$J_{IP_{3}R}=\frac{I_{IP_{3}R}}{F\cdot z\cdot A_{IP_{3}R}}\cdot \frac{\left(C_{s}-C_{C}\right)}{\left(C_{s,0}-C_{C,0}\right)}$$

The SERCA pumps are considered as bidirectional. We used the same kinetic expression as McIvor et al. (32). As in the latter study, we did not include Ca^2+^ buffering explicitly, given both the short time scales considered and the fact that local Ca^2+^ buffers are near saturation given the high Ca^2+^ concentrations reached around the channel mouths. Values of parameters are taken from McIvor et al. (32) and indicated in **Table S1**. For the processes that were not considered in this previous study, parameter values are discussed in the text and indicated in the figure legends or in the **Supplementary Information**.

To solve the partial differential equations (PDE), we used the finite element method (FEM) and simulation software COMSOL Multiphysics 5.5 (http://www.comsol.com) instead of using Green’s functions as it was done in McIvor et al. (32). This choice is based on the considerable extra flexibility and accuracy provided by the software, since it includes a specific physics-controlled domain discretization and solver configuration, which permits geometry modifications or model expansions. The computational capacity of the software also allowed us to rapidly perform a large number of simulations to explore various possible biological situations.

## Results

### Are ORAI1 Channel Openings Sufficient to Explain Non-TCR/CD3-Dependent Ca^2+^ Microdomains Mechanistically?

**Figure 2:**
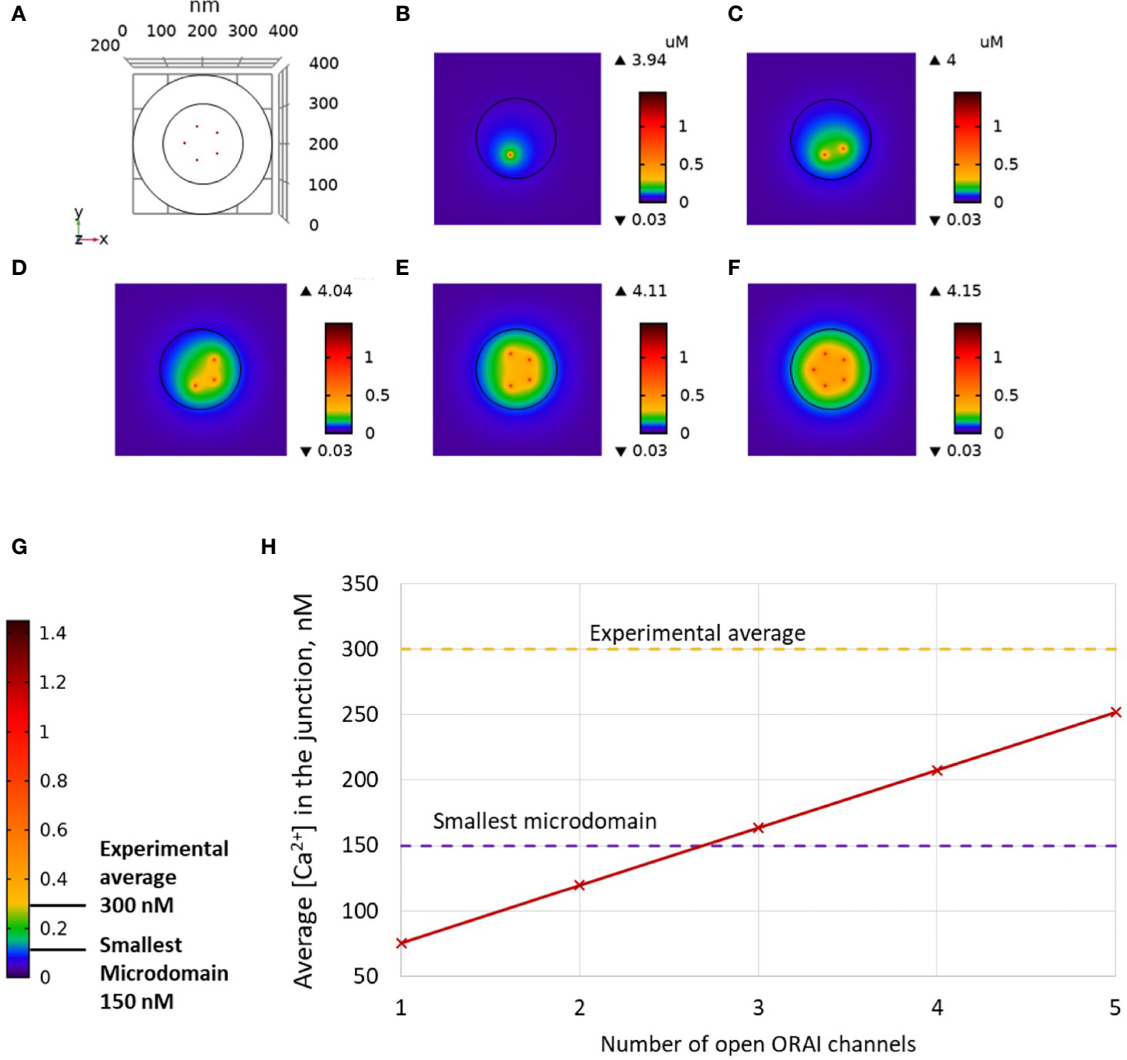
Simulated Ca^2+^ microdomains resulting from the opening of ORAI1 channels under the conditions of a full ER. **(A)** Upper view of the arrangement of the ORAI1 channels on the PM of the junction using COMSOL. **(B–F)** Steady-state Ca^2+^ profiles in the junction when opening 1 **(B)** to 5 **(F)** ORAI1 channels simultaneously. Left bars indicate the color codes, together with the minimal and maximal concentrations reached in the related panels. Shown are the profiles 22ms after opening of the ORAI1s, but these stabilize very rapidly, after a few ms. **(G)** Extended color code with marking of the smallest amplitude experimentally considered to correspond to a microdomain and of the average amplitude of microdomain in unstimulated T cells (4). **(H)** Evolution of the amplitude of the simulated Ca^2+^ microdomains with the number of simultaneously open ORAI1 in the junction, showing that experimentally observed microdomains cannot result from the sole opening of the ORAI1 in conditions of a full ER that only allows for a partial opening of these channels (see text).

Non-TCR/CD3-dependent Ca^2+^ microdomains last for 44 ± 4 ms and reach amplitudes of 290 ± 12 nM (4). Further, such Ca^2+^ microdomains were not decreased in T cells devoid of RYR1 (4). To determine whether ORAI1 channel openings are sufficient to explain non-TCR/CD3-dependent Ca^2+^ microdomains mechanistically, in a first approach, we used our model to predict the amplitude and spatial extent of the changes in junctional [Ca^2+^] induced by the opening of ORAI1 channels, in the presence of SERCA pumps as schematized in **Figure 1**. IP_3_Rs are not considered in these first simulations. The ER is assumed to be full to reproduce the situation of unstimulated T cells in which Ca^2+^ microdomains have been observed. In consequence, ORAI1 channels open at 21% of their full current when attached to Ca^2+^-unbound STIM2 (**Figure S1**). This situation is assumed to correspond to the spontaneous opening of an ORAI1 channel in the conditions of a full ER, mediated by a pre-formed ORAI1-STIM2 complex from which ER Ca^2+^ spontaneously dissociates due to a random fluctuation. In **Figure 2**, we considered an opening duration of 44 ms, based on the observed lifespan of the microdomains. Opening of one ORAI1 channel leads to a Ca^2+^ increase up to 4 μM at the cytosolic mouth of the channel. Because of the steep Ca^2+^ gradient across the PM, Ca^2+^ increases nearly instantaneously at the channel mouth. When ORAI1 closes, the microdomain disappears as soon as ORAI1 closes, given the small size of the domain respective to the cytosol. The spatial extent around the channel mouth is approx. 40-50 nm in diameter, which much exceeds the dimension of the channel pore, due to diffusion. However, this spatial extent remains significantly below the optical resolution of most current live cell imaging systems, preventing meaningful comparison with experimental data. The amplitude of the average Ca^2+^ increase in the junction is however below the experimentally determined lower limit (**Figures 2C, H**). From these computational results, one can conclude that the experimentally observed microdomains do not correspond to the opening of one ORAI1 channel in the conditions of full ER. If three or more ORAI1 open simultaneously, this creates a Ca^2+^ signal just below the plasma membrane that can be classified as a microdomain, as defined by Diercks et al. (4). For five open channels, the Ca^2+^ signal has an amplitude of 252 nM and a spatial extent of 0.04 μm^2^. It is worth noticing that five ORAI1 channels simultaneously open at 21% during 44 ms correspond to the entry of ~300 Ca^2+^ ions in the junction. However, given the small volume of the junction and the high diffusion coefficient of free Ca^2+^ (220 μm^2^/s), the time of residence of an ion in the junction is of the order of 0.045 ms. Because of this small residence time and of the activity of SERCA pumps, average numbers of free Ca^2+^ ions in the microdomain evaluated on the basis of the simulated concentrations thus remain smaller than one.

The Ca^2+^ signals created by the simultaneous opening of five ORAI1 channels are thus not compatible with the average amplitude of 300 nM observed experimentally. This indicates that even in the unlikely situation of a simultaneous opening of the five ORAI1 channels of the junction in conditions of a full ER, the amount of Ca^2+^ entering in the simulated junction is too small to account for experimental observations (Anim. S1). Indeed, SERCA pumps, which may in principle attenuate the Ca^2+^ increase, have a very limited activity in these conditions. The maximal Ca^2+^ concentration at the ER mouth of simulated pumps does not exceed 402 μM. In conclusion, simulations of the Ca^2+^ fluxes in and out the PM-ER junction show that the spontaneous opening of ORAI1 channels in a conductance state that corresponds to a full ER cannot account for the non-TCR/CD3-dependent Ca^2+^ microdomains observed in T cells.

### Concerted Activity of IP_3_R and ORAI1 Channels Mimics Non-TCR/CD3-Dependent Ca^2+^ Microdomains

Junctional small-scale Ca^2+^ increases thus probably involve an ER-related component. RYRs are not significantly involved in the formation of non-TCR/CD3-dependent microdomains in T cells (4). We thus explored the possible involvement of IP_3_Rs. It is indeed well known that in resting cells, some IP_3_R can open in the presence of the basal IP_3_ concentration to release luminal Ca^2+^ and create short-lived blips or puffs of Ca^2+^ in the cytoplasm (13, 42). Thillaiappan et al. (34) have shown that Ca^2+^ puffs occur nearly exclusively at immobile puncta close to the PM and proposed that these IP_3_Rs may provide local ER depletion closest to the PM where SOCE can occur. We thus reasoned that Ca^2+^ microdomains in T cells may be associated with the opening of immobile IP_3_Rs located close to the junctions, either spontaneously or due to receptors other than TCR/CD3. Indeed, evidence for integrin evoked IP_3_ was obtained recently (15). In this scenario, microdomains would result in part from Ca^2+^ flowing through the IP_3_Rs located near the junction and in another part from the ORAI1-mediated Ca^2+^ entry that follows the local, IP_3_-induced decrease in ER Ca^2+^.

**Figure 3:**
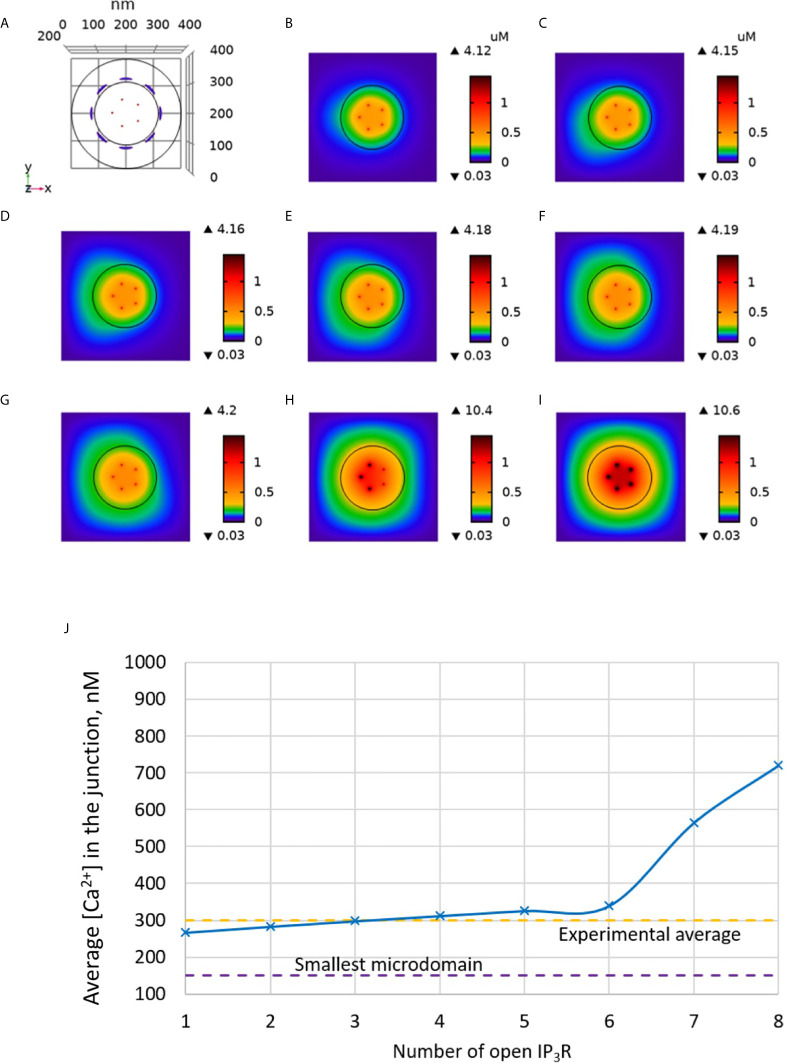
Simulated Ca^2+^ microdomains resulting from the opening of the IP_3_Rs adjacent to the junctions, which in turn induces the opening of ORAI1 channels in the junctions as a result of local depletion of ER Ca^2+^. **(A)** Upper view of the arrangement of the ORAI1 channels on the PM of the junction (red dots) and of the adjacent IP_3_Rs (blue lines) using COMSOL. **(B–I)** Steady-state Ca^2+^ profiles in the junction when opening 1 **(B)** to 8 **(I)** IP_3_Rs simultaneously. Left bars indicate the color codes, together with the minimal and maximal concentrations reached in the related panel. Shown are the profiles 22ms after opening of the IP_3_Rs. Upon depletion of local Ca^2+^ in the ER, which is quasi-instantaneous, ORAI1 channels open to an extent that depends on this local concentration, as defined by the function *f* (see **Supplementary Information**). ORAI1 opening is assumed to occur immediately after depletion because ORAI1-STIM2 aggregates are pre-formed (4). **(J)** Evolution of the amplitude of the simulated Ca^2+^ microdomains with the number of simultaneously open IP_3_Rs in the junction, showing that experimentally observed microdomains can in principle result from the opening of ORAI1 channels induced by the spontaneous opening of a few IP_3_Rs near the junction, in conditions of a full ER.

**Figure 4:**
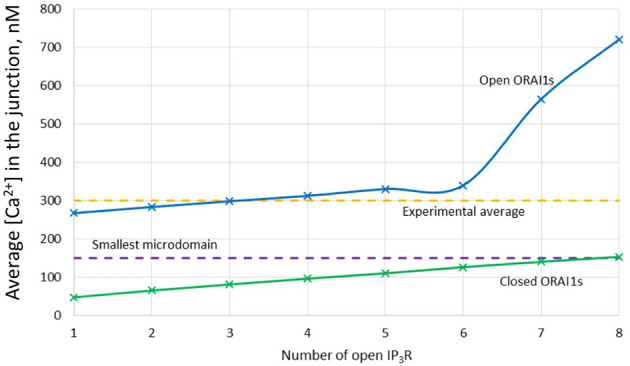
Evolution of the amplitude of the simulated Ca^2+^ microdomains with the number of simultaneously open IP_3_Rs in the junction in the presence and in the absence of ORAI1s in the junction. The blue curve (with ORAI1) corresponds to the situation considered in **Figure 3**. The theoretical situation of a junction that does not contain ORAI1 channels (green curve) allows to appreciate that the contribution of Ca^2+^ released through the IP_3_Rs to the Ca^2+^ microdomain is rather limited.

We tested this hypothesis computationally by including IP_3_Rs in the model, as schematized in **Figure 1**. In **Figure 3** increasing numbers of IP_3_Rs are opened during 44 ms, to simulate the stochastic opening of these receptors in the presence of a basal concentration of IP_3_. ORAI1 channels then open according to the ER Ca^2+^ concentration around the closest IP_3_Rs. In this situation, Ca^2+^ microdomains with realistic amplitudes and invading the whole junction are created when 2 to 5 IP_3_Rs are assumed to open simultaneously (Anim. S2a). Clearly, two sources of Ca^2+^ contribute to this Ca^2+^ event: first, Ca^2+^ released by nearby IP_3_Rs can diffuse in the junction as discussed below. Second, the local depletion at the luminal extremity of the IP_3_R activates ORAI1 opening and Ca^2+^ influx from the extracellular medium. Because in the luminal region surrounding an open IP_3_R, ER Ca^2+^ drops to 330 μM, closest ORAI1 channels open half-maximally (**Figure S1**), generating a Ca^2+^ flux through ORAI1 that is more than twice the one occurring in the absence of IP_3_R. The combination between the Ca^2+^ fluxes through the IP_3_R and ORAI1 allow the formation of a Ca^2+^ microdomain in the junction that agrees with observations in T cells. **Figure 6A** shows that the Ca^2+^ concentration at the cytosolic side of the IP_3_R reaches ~16 μM (Anim. S3a, S4a, S5a & S6a). However, IP_3_Rs are not in the junction itself and the contribution of ER Ca^2+^ in Ca^2+^ microdomains is rather limited, as attested by simulations that do not consider Ca^2+^ entry (**Figure 4**). The local depletion created by the InsP_3_-induced Ca^2+^ release however plays a crucial role in allowing ORAI1 to open to a larger extent than with a full ER. As visible in **Figure 3**, when more than 6 IP_3_Rs open simultaneously, the Ca^2+^ increase in the domain much exceeds those observed experimentally. This is in agreement with the fact that only a fraction of IP_3_Rs can spontaneously open at basal IP_3_ concentration. Thus, non-TCR/CD3-dependent Ca^2+^ microdomains such as those observed in T cells can be reproduced with the model when considering both the ORAI1 channels located in the junction and the adjacent IP_3_Rs.

### The Diffusion Coefficient of Ca^2+^ in the ER Dramatically Affects the Amplitude of Non-TCR/CD3-Dependent Ca^2+^ Microdomains

**Figure 5:**
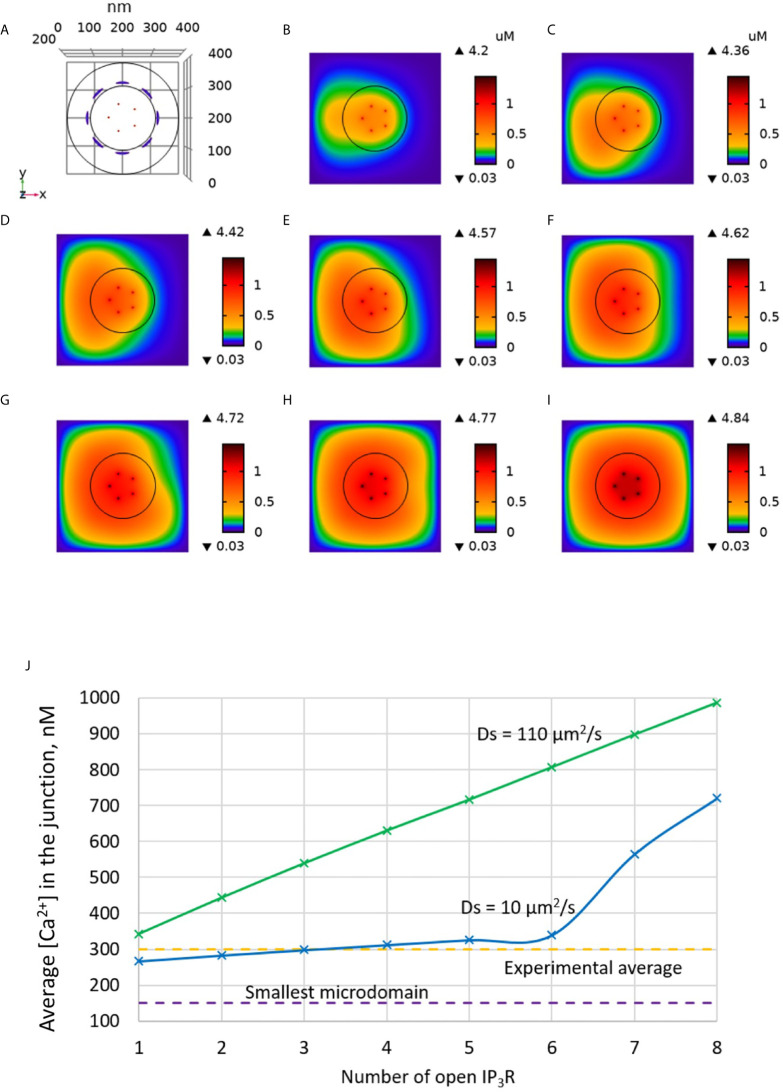
Influence of the value of the Ca^2+^ diffusion coefficient in the ER (D_S_) on the Ca^2+^ microdomains in the ER-PM junction. **(A)** Upper view of the arrangement of the ORAI1 channels on the PM of the junction (red dots) and of the adjacent IP_3_Rs (blue lines) using COMSOL. **(B–I)** Steady-state Ca^2+^ profiles in the junction when opening 1 **(B)** to 8 **(I)** IP_3_Rs simultaneously with D_S_ = 110 μm^2^/s. Left bars indicate the color codes, together with the minimal and maximal concentrations reached in the related panels. Shown are the profiles 22ms after opening of the IP_3_Rs. Upon depletion of local Ca^2+^ in the ER, which is quasi-instantaneous, ORAI1 channels open to an extent that depends on this local concentration, as defined by the function *f* (see **Supplementary Information**). ORAI1 opening is assumed to occur immediately after depletion because ORAI1-STIM2 aggregates are pre-formed (4). **(J)** Evolution of the amplitude of the simulated Ca^2+^ microdomains with the number of simultaneously open IP_3_Rs in the junction. Results obtained with the default value for D_S_ (10 μm^2^/s) corresponding to the results shown in **Figure 3** are also indicated for comparison.

**Figure 6:**
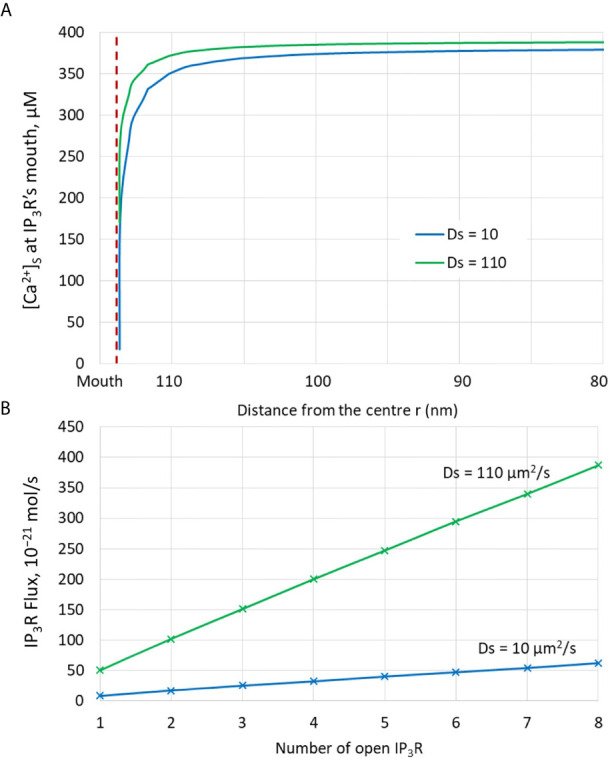
Analysis of the influence of the presence of IP_3_Rs and of the value of the Ca^2+^ diffusion coefficient in the ER, D_S_. **(A)** Ca^2+^ concentration around the luminal mouth of an IP_3_R for the two values of D_S_ considered in the simulations. The largest this value, the fastest the replenishment around an open channel. **(B)** Fluxes through open IP_3_Rs for the two values of D_S_ considered in the simulations. Because of faster replenishment around an open channel when D_S_ is larger, the concentration gradient around the two extremities of the channel pore is larger, and hence the flux.

**Figure 7:**
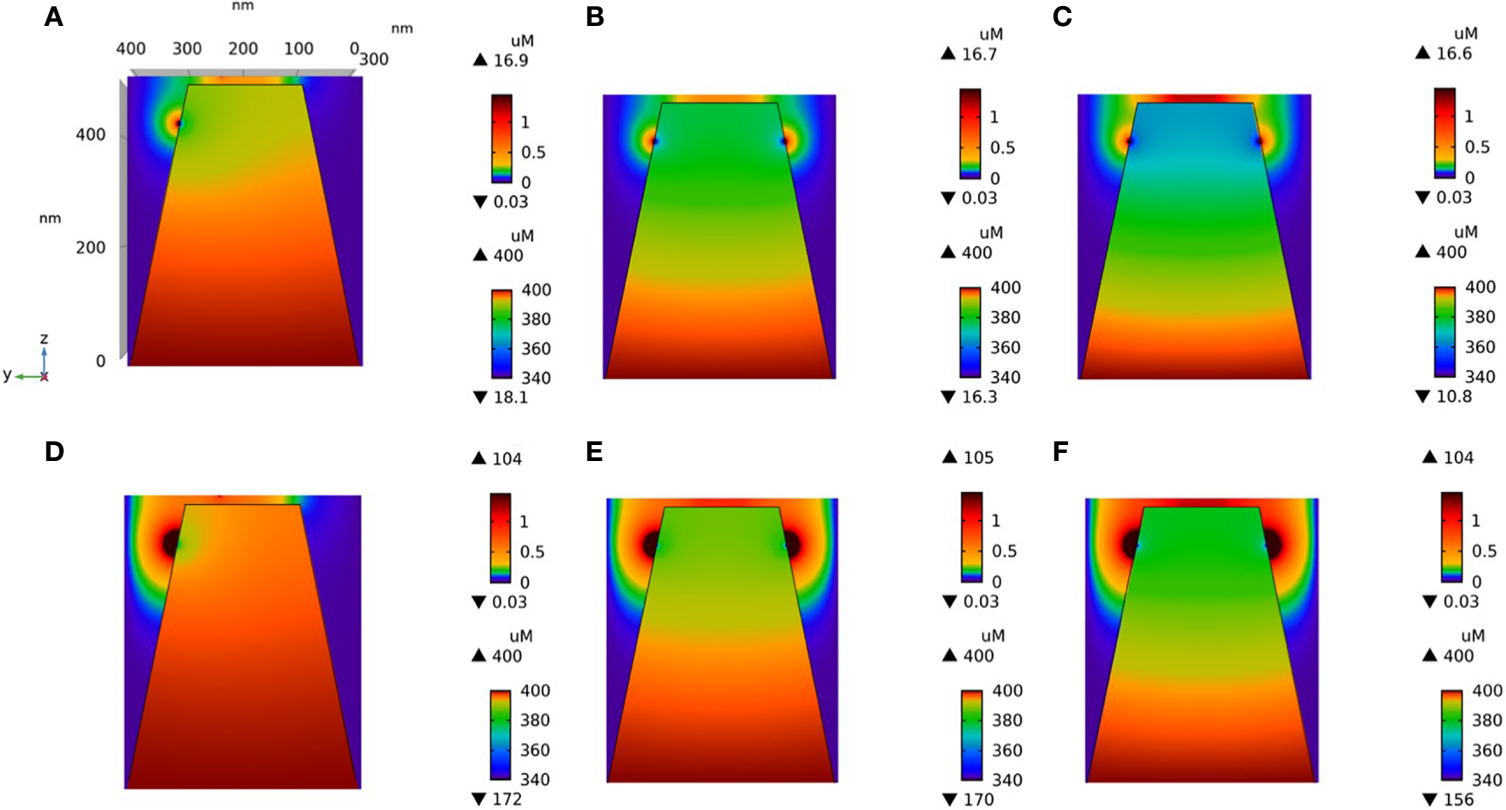
Cross-section of the Ca^2+^ profiles in the junction, in the cytosol adjacent to the junction and in the sub-PM ER during microdomain for two values of the Ca^2+^ diffusion coefficient in the ER, D_S_. **(A–C)**: microdomains created by the opening of 2, 5 and 7 IP_3_Rs, respectively, for D_S_ = 10 μm^2^/s. Local depletion of ER Ca^2+^ provokes the opening of the nearby ORAI1s. This situation corresponds to the one shown in **Figure 3**. **(D–F)**: microdomains created by the opening of 2, 5 and 7 IP_3_Rs, respectively, for D_S_ = 110 μm^2^/s. Local depletion of ER Ca^2+^ provokes the opening of the nearby ORAI1s. This situation corresponds to the one shown in **Figure 5**. For all panels, the upper right bar indicates the color code of Ca^2+^ concentration in the cytosol while the lower right bar indicates the color code of Ca^2+^ concentration in the ER.

Much uncertainty remains as to the exact value of the diffusion coefficient of Ca^2+^ in the ER (21, 32, 43, 44). We thus took profit of the possibility offered by computational modelling to analyze the effect of changing the value of this key parameter. Increasing D_S_ from 10 (43, 44) to 110 (21) has a drastic effect on the dynamics of junctional Ca^2+^. Such a change could be related to a decrease in the buffering capacity of the ER, or to a change in the tortuosity that characterizes this organelle. As seen in **Figure 5**, opening of one IP_3_R with a high value of D_S_ suffices to create an extended microdomain whose amplitude exceeds the experimental average junctional amplitude. Interestingly, simulations show that in this case, ORAI1 channels never open in a more than minimal conductance state because the Ca^2+^ concentration in a region surrounding an open IP_3_R does not drop below 360 μM, which is larger than the 330 μM reached in the same volume when D_S_=10 μm^2^/s (Anim. S2b). Changes in the value of the diffusion coefficient much affects the Ca^2+^ profile near the mouth of the channel (**Figure 6A**). When the diffusion coefficient increases, Ca^2+^ is replenished faster. This does not only affect the state of ORAI1, but also the flux through the IP_3_R. The concentration difference between the luminal and cytosolic extremities of the channel is indeed larger than in the slow diffusion situation, which increases the Ca^2+^ flux (**Figure 6B**). Such effect is also visible in **Figure 7** where the Ca^2+^ profiles in the presence of ORAI1 channels can be compared for the two values of the diffusion coefficient (Anim. S3b, S4b, S5b & S6b). Simulations thus predict that if Ca^2+^ diffusion inside the ER is fast (D_S_ = 110 μm^2^/s), non-TCR/CD3-dependent Ca^2+^ microdomains could occur in the absence of extracellular Ca^2+^ or in T cells that do not express ORAI1, which does not agree with experimental observations (4).

### ORAI1 Channel Clustering Without Major Effect On Non-TCR/CD3-Dependent Ca^2+^ Microdomains

**Figure 8:**
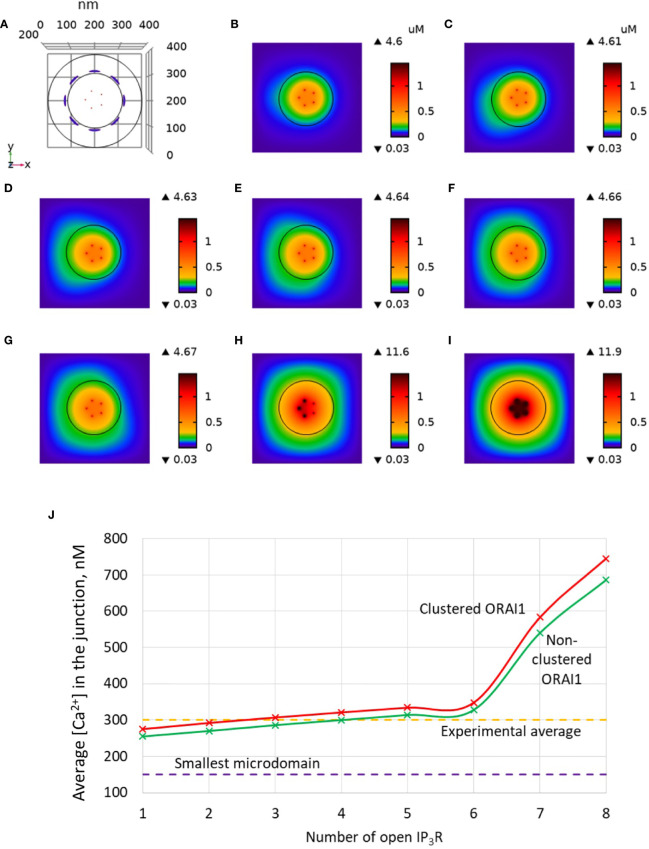
Influence of the distance between ORAI1 channels on the Ca^2+^ microdomains in the ER-PM junction. The situation is similar to the default situation shown in **Figure 3**, except for the distance between ORAI1 channels that is here equal to 37.7 nm (clustered) instead of 47 nm (non-clustered). **(A)** Upper view of the arrangement of the ORAI1 channels on the PM of the junction (red dots) and of the adjacent IP_3_Rs (blue lines) using COMSOL. **(B–I)** Steady-state Ca^2+^ profiles in the junction when opening 1 **(B)** to 8 **(I)** IP_3_Rs simultaneously. Left bars indicate the color codes, together with the minimal and maximal concentrations reached in the related panel. Shown are the profiles 22ms after opening of the IP_3_Rs. Upon depletion of local Ca^2+^ in the ER, which is quasi-instantaneous, ORAI1 channels open to an extent that depends on this local concentration, as defined by the function *f* (see **Supplementary Information**). ORAI1 opening is assumed to occur immediately after depletion because ORAI1-STIM2 aggregates are pre-formed (4). **(J)** Evolution of the amplitude of the simulated Ca^2+^ microdomains with the number of simultaneously open IP_3_Rs in the junction. Results obtained with the default value of the inter-ORAI1 channels distance (47 nm, non-clustered) corresponding to the results shown in **Figure 3** are also indicated for comparison.

**Figure 9:**
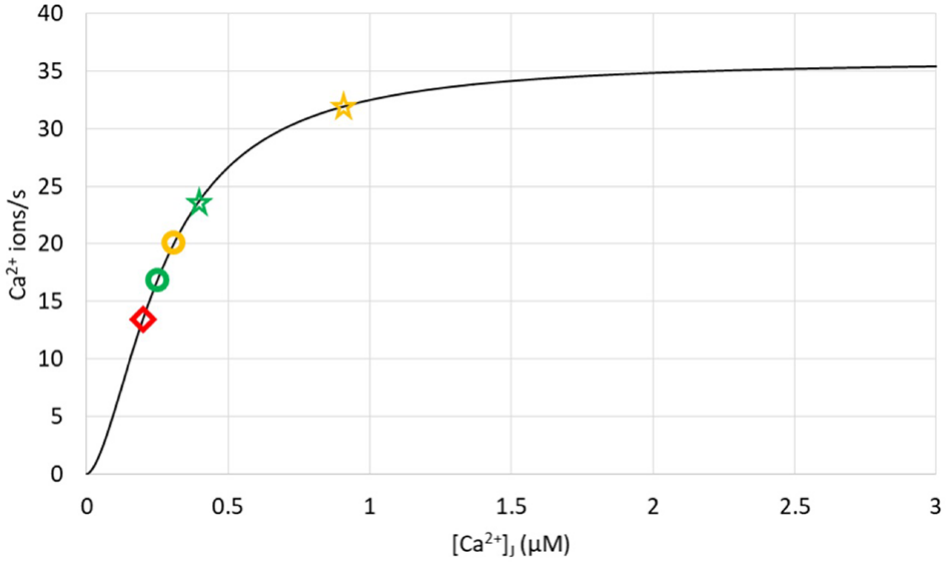
Ca^2+^ fluxes through SERCA pumps in different simulation conditions showing that pumps are not saturated during Ca^2+^ microdomains. The curve represents the rate of pumping as a function of cytosolic Ca^2+^, as given by Eq. S19, with C_S_ = 400 μM. Yellow symbols correspond to the opening of the 4 IP_3_Rs nearest to the SERCA, with the circle corresponding to D_S_ = 10 μm^2^/s and the star to D_S_ = 110 μm^2^/s. Green symbols correspond to the opening of the 4 IP_3_Rs furthest from the SERCA, with the circle corresponding to D_S_ = 10 μm^2^/s and the star to D_S_ = 110 μm^2^/s. The red diamond refers to a situation with closed IP_3_Rs, 5 ORAI1 channels of the junction being open as in **Figure 2**.

Another factor that may have an influence on the amplitude of the Ca^2+^ microdomain is the distance between ORAI1 channels in the ER-PM junction. This was investigated in our simulations by decreasing the distance between ORAI1 channels from 47 to 37.7 nm. As shown in **Figure 8**, channel clustering has a very limited effect on the characteristics of the Ca^2+^ microdomains, resulting in a slight increase in the amplitude of the Ca^2+^ signal in the junction. This conclusion does not depend on the value of the diffusion coefficient in the ER (not shown). Actually, clustering of the ORAI1 channels could increase the amplitude of the microdomain if either Ca^2+^ diffusion in the junction or Ca^2+^ pumping was affected by the inter-channel distance. Since Ca^2+^ increases near ORAI1 channels always remain highly localized in both situations, diffusion is not much affected. SERCA pump activity associated with an active microdomain based on clustered or non-clustered ORAI1 channels are shown in **Figure 9**. Steady-state Ca^2+^ fluxes through SERCA pumps when IP_3_Rs and ORAI1 channels are open are clearly not much sensitive to the conditions, whatever the value of the Ca^2+^ diffusion coefficient in the ER. Interestingly, pumps are not saturated in contrast to the situation encountered with a highly depleted ER (32).

## Discussion

Ca^2+^ signaling is fundamental for activation of T cells, where it largely relies on SOCE. Although SOCE controls physiological functions at the cellular level, it creates highly heterogeneous local Ca^2+^ signals. Given that Ca^2+^ channels, pumps and downstream signaling molecules are controlled locally, cellular Ca^2+^ dynamics results from the detailed regulation of SOCE in an ensemble of compartmentalized microdomains.

In the present modeling study, we have investigated Ca^2+^ dynamics in a confined configuration corresponding to a junctional cytosolic space and the adjacent sub-PM ER, taking into account Ca^2+^ influx through ORAI1 and IP_3_R, Ca^2+^ pumping into the ER through SERCA, and diffusion within the cytosolic and ER compartments. Our main aim was to gain insight into the mechanistic origin of the Ca^2+^ microdomains observed in non TCR/CD3 stimulated T cells that have been reported in Diercks et al. (4). These are short-lived (<50 ms), low amplitude (~300 nM) Ca^2+^ increases located just below the PM. Their spatial spread is most probably below the spatial resolution of the optical imaging system, i.e. 368 nm. Importantly, they do not involve Ca^2+^ release through RYR1, but we obtained initial evidence for a signaling pathway evoked by weak cell adhesion to β1-integrins, IP_3_ signaling, and ORAI activation *via* pre-formed complexes with STIM1 and STIM2 (15). Weak adhesion of T cells migrating to inflamed tissue occurs during leukocyte rolling in venules, during diapedesis and migration through basement membrane and interstitium. Thus, such weak adhesion processes during the T cell’s journey into the inflamed area may lift the activation status from a non-activated, quiescent cell to a pre-activated T cell. This shift in activation state would then facilitate full activation through the TCR/CD3 complex by local antigen-presenting cells.

For that purpose, we modified several aspects of the spatial model of SOCE previously proposed by McIvor et al. (32). First, we considered the situation of a full ER with C_S_ = 400 μM, while the former study focused on a massively depleted ER, i.e. C_S_ = 150 μM. This change affects initial and boundary conditions, but also led us to consider the sub-maximal conductance states of ORAI1 corresponding to not fully STIM-bound ORAI channels (37, 45, 46). We indeed hypothesized that a larger C_S_ results in a reduced availability of Ca^2+^-unbound STIM that are susceptible to bind to and open ORAI1. This assumption agrees with the observed relationship between the SOC current and C_S_ reported at the whole cell level (7, 10). Second, we took into account the presence of IP_3_Rs in regions of the ER membrane close to the junctional space, following a spatial arrangement suggested by Thillaiappan et al. (34). As a consequence, we also considered a slightly larger portion of the cell than McIvor et al. (32). From a technical point of view, we resorted to COMSOL Multiphysics, which allowed us extra flexibility and considerably reduced the computing time. This software not only allows to simulate realistic geometries but is also very well adapted to the computational study of Ca^2+^ dynamics in confined domains in which steep concentrations gradients arise, as for example in dendritic spines (47). Additionally, it allows to easily include, remove or spatially rearrange the model’s components (like pumps, channels or Ca^2+^ stores) in order to match experimental conditions, as it was done by Maccari et al. (48) to show the role of mitochondria relocation and PMCA pumps accumulation at the immunological synapse in global Ca^2+^ increase during T cell polarization.

**Figure 10:**
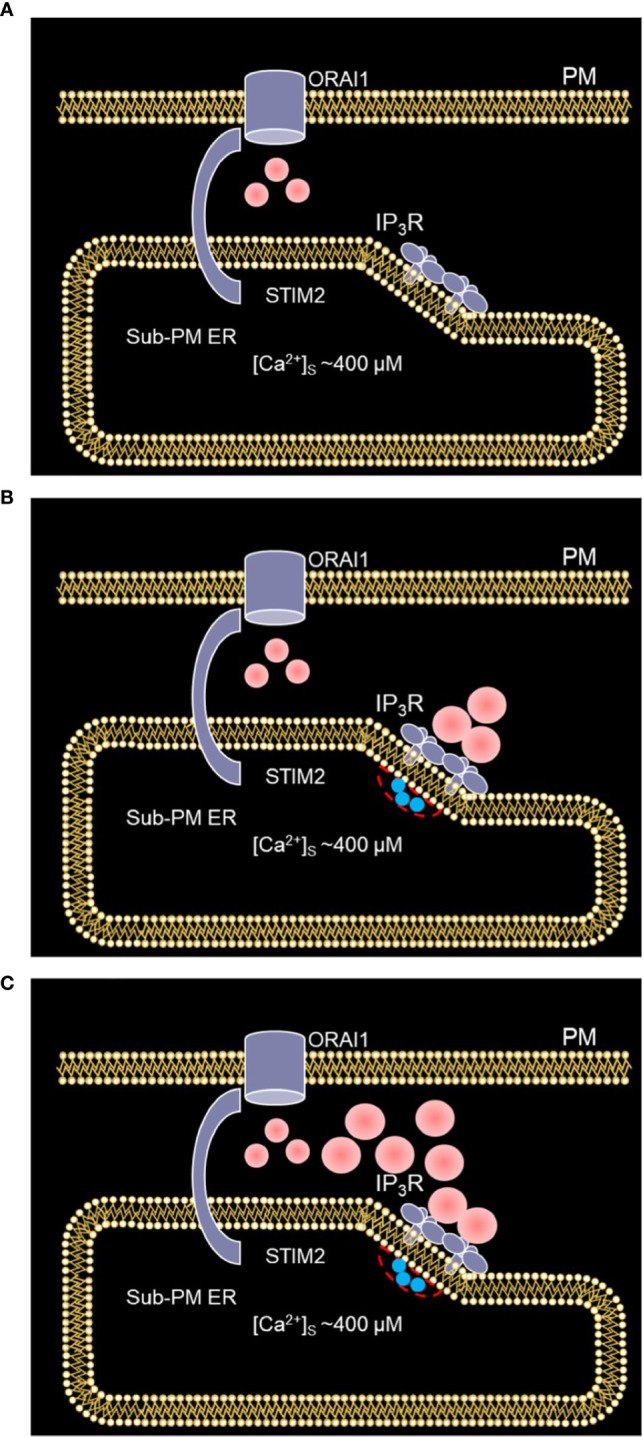
Schematized representation of the proposed mechanism underlying the spontaneous formation of Ca^2+^ microdomains in T cells. **(A)** The smallest microdomains arise from nano-scale [Ca^2+^] fluctuations in the sub-PM ER leading to the opening of ORAI1 channels STIM2 inherently co-localized with STIM2. **(B)** Following a short, spontaneous activation of one or a few IP_3_Rs close to the junction, Ca^2+^ is released from the sub-PM ER into the cytosol. **(C)** The resulting local Ca^2+^ depletion close to the IP_3_R’s mouth provokes the unbinding of Ca^2+^ from STIM2, which further activates ORAI1 channels. This results in larger Ca^2+^ signals in the microdomains. Red spots represent Ca^2+^ ions.

Based on our simulations, we concluded that the concerted activity of IP_3_R and ORAI1 channels is responsible for non-TCR/CD3 dependent Ca^2+^ microdomains in T cells *via* the mechanism schematized in **Figure 10**, where two different states are described. In a first state (**Figure 10A**), nano-scale [Ca^2+^] fluctuations in the sub-PM ER (25) are sensed by STIM2 inherently co-localized with ORAI1 (7, 15). The latter ORAI1 channels are then activated shortly and partially (37) and form small microdomains. Although it is likely that these ORAI1-based microdomains correspond to some of the events observed in the absence of TCR/CD3 stimulation, they cannot account on their own for all the events reported since even 5 simulated ORAI1 channels simultaneously open in the junction do not increase [Ca^2+^] up to a level that corresponds to the experimental average [Ca^2+^]. In a second state (**Figure 10B**), following a short activation of one or a few immobile IP_3_Rs close to the junction (34), Ca^2+^ is released through IP_3_Rs from the sub-PM ER into the cytosol. The resulting local Ca^2+^ depletion close to the IP_3_R’s mouth provokes the unbinding of Ca^2+^ from STIM2, which further activates ORAI1 channels (**Figure 10C**) increasing Ca^2+^ in the junction and thus forming larger non-TCR/CD3-dependent Ca^2+^ signals in the microdomains. Although it is important that the IP_3_Rs are located close to the junction to create local depletions that can be sensed by SOC channels, simulations suggest that these receptors are not located inside the junction (34). Indeed, if this would be the case, their opening would generate an increase in junctional Ca^2+^ concentration that would much exceed the observed amplitude of a microdomain. Such a computational observation is expected given the respective values of the currents through ORAI1 and IP_3_Rs.

We also used the model to investigate the impact of the distance between ORAI1 channels in the junction. In agreement with the results of McIvor et al. (32), we found that ORAI1 clustering has a limited effect on the characteristics of the Ca^2+^ microdomain, both in the presence and in the absence of IP_3_Rs. Clustered ORAI1 channels lead to higher local [Ca^2+^], an effect that is compensated by the reduced extent of the Ca^2+^ increase. In contrast, the value of the Ca^2+^ diffusion coefficient in the ER (D_s_) has a drastic effect on the Ca^2+^ microdomain in the junction. Agreement with observations could only be reached when considering D_s_ = 10 μm^2^/s, i.e. a value that is ~20 times smaller than the value reported for the cytoplasm. This smaller value could result from molecular crowding in the lumen (43) and from the tortuosity of the tubular network of the ER (49). The latter effect is expected to be particularly strong in the sub-PM regions of the ER adjacent to the junctions.

At this stage, the contribution of IP_3_Rs has been included in a simple way. It is well established that the opening of these channels is stochastic and regulated by cytosolic Ca^2+^ (17, 50), two factors that need to be considered in a refined version of the model. In particular, we have shown here that local depletion of C_S_ following the opening of an IP_3_R favors the opening of ORAI1. However, resting STIMs negatively regulate IP_3_R’s activity (51). Moreover, the entry of Ca^2+^ through ORAI1 could further activate Ca^2+^ release through the IP_3_R. These two feed-forward loops are expected to be particularly significant when IP_3_ is produced, as it is the case upon the intermediate phase of TCR/CD3 stimulation, e.g. after the initial phase of NAADP/RYR1 signaling in the first 15 s (3, 4, 52).

Next, a quantitative model describing Ca^2+^ microdomains elicited by NAADP/RYR1 signaling, e.g. NAADP/RYR1-dependent increased number of microdomains and transition of Ca^2+^ microdomains into global Ca^2+^ signaling in the first 15 s of directed TCR/CD3 signaling, should be established. Such an extended model including a detailed description of the interplay between SOCE dynamics, NAADP signaling, RYR1s and IP_3_Rs should improve our detailed understanding of the first steps of T cell activation.

## Data Availability Statement

The original contributions presented in the study are included in the article/**Supplementary Material**. Further inquiries can be directed to the corresponding author.

## Author Contributions

DG, AG and GD contributed to the conceptualization and design of the study. DG, AG and GD contributed to the development and analysis of the model. DG performed all numerical simulations. All authors contributed to the article and approved the submitted version.

## Funding

This work was supported by the Deutsche Forschungsgemeinschaft (DFG) (project number 335447717; SFB1328, project A01 to AG), by the Joachim-Herz-Stiftung (Hamburg), Infectophysics Consortium (project 4; to AG), by NCL-Stiftung Hamburg (to AG), the Hamburg Ministry of Science, Research and Equality (LFF- FV75/0070-134, to AG), and by University Medical Center Hamburg-Eppendorf (M3I consortium, to AG). Research in the AG labs is also supported by EU project INTEGRATA - DLV-813284. GD is Research Director at the FNRS.

## Conflict of Interest

The authors declare that the research was conducted in the absence of any commercial or financial relationships that could be construed as a potential conflict of interest.
