# Supplementary material for: Three-Dimensional Model of Sub-Plasmalemmal Ca2+ Microdomains Evoked by the Interplay Between ORAI1 and InsP3 Receptors
Source: Front Immunol. 2021 Apr 28;12:659790. doi: 10.3389/fimmu.2021.659790 (PMC8113648; doi:10.3389/fimmu.2021.659790)
Supplement: Supplementary file 1 [file DataSheet_1.pdf]

Three-dimensional model of sub-plasmalemmal Ca^{2+} microdomains evoked by the interplay between ORAI1 and $InsP_3$ receptors

Diana Gil[1], Andreas H. Guse [1] and Geneviève Dupont[2]

[1] The Ca^{2+} Signalling Group, Department of Biochemistry and Molecular Cell Biology, University Medical Center Hamburg-Eppendorf, Martinistraße 52, 20246 Hamburg, Germany

[2] Unit of Theoretical Chronobiology, Faculté des Sciences CP231, Université Libre de Bruxelles (ULB), Boulevard du Triomphe, B-1050 Brussels, Belgium

Supplementary Information

Description of the mathematical model

Our mathematical model describes the evolution in time and space of free cytosolic Ca^{2+} concentration C_C= C_C(t,x,y,z) and of free ER Ca^{2+} concentration C_S= C_S(t,x,y,z), both in close proximity of the ER-PM junction; and governed by the diffusion equations S1 and S2 respectively,

$$\frac{\partial C_C}{\partial t} - D_C \nabla^2 C_C = 0 \qquad \text{(S1)},$$

$$\frac{\partial C_S}{\partial t} - D_S \nabla^2 C_S = 0 \qquad \text{(S2)},$$

together with the initial conditions $C_{C,0}$ and $C_{S,0}$:

$$C_{C,0} = 0.03\ \mu M \qquad \text{(S3)},$$

$$C_{S,0} = 400\ \mu M \qquad \text{(S4)}.$$

Ca^{2+} ions are transferred across domains through channels and pumps. The corresponding boundary conditions follow:

For C_C,

$$D_C \left.\frac{\partial C_C}{\partial z}\right|_{z=500nm} = \begin{cases} J_{ORAI}, & \text{at ORAI1 channels} \\ 0, & \text{rest of the PM,} \end{cases} \qquad \text{(S5)},$$

$$D_C \left.\frac{\partial C_C}{\partial z}\right|_{z=485nm} = \begin{cases} J_{SERCA}, & \text{at SERCA pumps} \\ 0, & \text{rest of the ERM,} \end{cases} \qquad \text{(S6)},$$

$$D_C \frac{\partial C_C}{\partial \phi}\bigg|_{\phi=\frac{5\pi}{6}} = \begin{cases} J_{IP_3R}, & \text{at IP}_3\text{R channels} \\ 0, & \text{rest of the sub} - \text{PM ER surface}, \end{cases} \quad \text{(S7)},$$

$$C_C|_{x,y\,\in\,\{0,400\}nm} = C_{C,0}. \quad \text{(S8)}.$$

For C_S,

$$D_S \frac{\partial C_S}{\partial z}\bigg|_{z=485nm} = \begin{cases} J_{SERCA}, & \text{at SERCA pumps} \\ 0, & \text{rest of the ERM}, \end{cases} \quad \text{(S9)},$$

$$D_S \frac{\partial C_S}{\partial \phi}\bigg|_{\phi=\frac{5\pi}{6}} = \begin{cases} J_{IP_3R}, & \text{at IP}_3\text{R channels} \\ 0, & \text{rest of the sub} - \text{PM ER surface}, \end{cases} \quad \text{(S10)},$$

$$C_S|_{z=0} = C_{S,0}. \quad \text{(S11)}.$$

The influx across one ORAI1 channel per unit area (Eq. S12) follows Faraday's law with the magnitude of a single channel current $I_{ORAI} = 2.1\ fA$, determined experimentally by Hoth & Penner (1992), *F* the Faraday constant, *z the* charge of a Ca^{2+} ion and the surface of the channel pore $A_O = 0.25\ nm^2$ observed by Parekh & Putney (2005)

$$J_{ORAI} = \frac{I_{ORAI}}{F \cdot z \cdot A_o} \cdot f\left(C_S^{loc}\right) \cdot \phi_{ORAI} \quad \text{(S12)},$$

multiplied by $f\,(C_S^{loc})$, a function of the average local concentration of luminal Ca^{2+} around the mouth of the IP_3R (Eq. S13), computed in a 108 nm^3 volume surrounding each IP_3Rs at the side of the ER. In the simulations, this concentration determines the level of ORAI1 channel activation, since it was suggested by Li et al. (2011) that this activation is not an "all or none" phenomenon but rather a graded process of four conductance states, which depends on the amount of bounded STIM proteins determined by the level of ER depletion,

$$f\left(C_S^{loc}\right) = \begin{cases} 0.07, & C_S^{loc} > 450\ \mu M \\ 0.21, & 450\ \mu M \leq C_S^{loc} < 334\ \mu M \\ 0.54, & 334\ \mu M \leq C_S^{loc} < 250\ \mu M \\ 1, & C_S^{loc} \geq 250\ \mu M \end{cases} \quad \text{(S13)}.$$

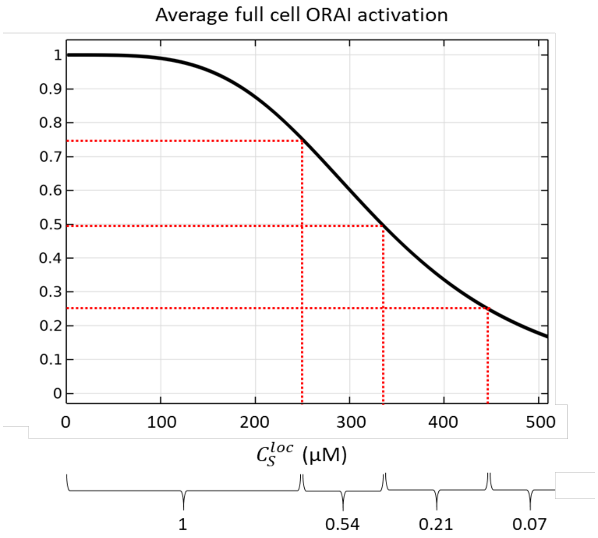

(Figure S1)

Given that we do not consider Ca^{2+}-bound STIM diffusion explicitly, we define the ranges of local luminal Ca^{2+} that correspond to each ORAI1 activation level. This assumption follows the observation of pre-formed clusters of ORAI and STIM (Diercks et al., 2018), allowing to consider that SOCE is immediately activated after Ca^{2+} unbinding from STIM. Function *f* defined in (S13) follows a fitted Hill function (Fig. S1) with K_D = 334 µM, which is twice the STIM1's dissociation constant observed experimentally by Luik et al. (2008), taking into account STIM2's lower Ca^{2+} affinity (Luik et al., 2008; Brandman et al., 2007).

An additional function Φ_{ORAI} multiplies the influx through one ORAI channel. It allows to simulate the two configurations of the model. The first one in which the IP_3R are closed and Ca^{2+} enters the cytosol only across ORAI1 channels and the second one where we consider open IP_3R,

$$\phi_{ORAI} = \begin{cases} \Pi(t)_i, & \text{closed IP}_3\text{R} \\ 1, & \text{open IP}_3\text{R} \end{cases} \qquad \text{(S14).}$$

In a similar way, the influx across one IP_3R is given by Eq. S15, with a single current magnitude of $I_{IP3R} = 0.064\ pA$ as estimated by Means et al. (2006) in correspondence to resting Ca^{2+} concentrations and $A_{IP3R} = 0.41\ nm^2$ as stated in Thul & Falcke (2004),

$$J_{IP_3R} = \frac{I_{IP_3R}}{F \cdot z \cdot A_{IP_3R}} \cdot \frac{(C_S - C_C)}{(C_{S,0} - C_{C,0})} \cdot \phi_{IP_3R} \qquad \text{(S15).}$$

The concentrations difference ($C_S - C_C$) is computed locally at each IP_3R and ($C_{S,0} - C_{C,0}$) stand for the resting concentrations difference. Given the time scales considered in the simulations, buffering is not considered. Once more we multiply the flux by a Φ_{IP3R} function (S16), which follows our two configurations,

$$\phi_{IP_3R} = \begin{cases} 0, & \text{closed IP}_3\text{R} \\ \Pi(t)_m, & \text{open IP}_3\text{R} \end{cases} \qquad \text{(S16).}$$

The rectangular function *Π(t)* changes in time between 0 and 1 deterministically and controls whether a channel is open or closed, accordingly with the respective configuration,

$$\Pi(t)_{\{i,m\}} = \Pi\left(\frac{t - \tau_{c_{\{i,m\}}}}{\tau_d}\right) \qquad \text{(S17),}$$

with $i \in \{1,2,3,4,5\}$ the corresponding ORAI1 channel and $m \in \{1,2,3,4,5,6,7,8\}$ the corresponding IP_3R depending on the configuration. The channels open during a time $\tau_d = 0.044\ s$. The central time $\tau_{c_{\{i,m\}}}$ when the channels are open is given by the following sequence:

$$\tau_{c\{i,m\}_n} = n * 0.1\ s + (n - 0.5) * 0.044\ s \quad \forall\, n \geq \{i, m\},\ \ n > 0 \qquad \text{(S18)},$$

where n increases gradually and represents the number of open channels and n stands for seconds.

We use bi-directional SERCA2b pumps as done by Shannon et al. (2004) with the respective kinetics parameters taken from Lytton et al. (1992), as done in McIvor et al. (2018),

$$J_{SERCA} = \frac{Q}{A_S} * V_{max} * \left[\frac{\left(\frac{C_C}{K_F}\right)^{H2b} - \left(\frac{C_S}{K_R}\right)^{H2b}}{1 + \left(\frac{C_C}{K_F}\right)^{H2b} + \left(\frac{C_S}{K_R}\right)^{H2b}} \right] \qquad \text{(S19)},$$

a summary of all parameters is given in Table S1.

We assume the evolution of Ca^{2+} concentration to be governed purely by isotropic diffusion, therefore we use an unbuffered cytosolic diffusion D_J=220 um^2/s (McIvor et al., 2018; Samanta et al., 2015; Hogan, 2015) considering that the high Ca^{2+} concentration at the mouth of the channels will saturate local Ca^{2+} buffers (Parekh, 2008). A fixed Ca^{2+} diffusion coefficient in the ER lumen remains yet to be determined, although it is known to be smaller than in the cytosol as a result of its irregular and cramped nature. A luminal diffusion coefficient including the effect of buffers has been measured to be around D_S=10 um^2/s (Dayel et al., 1999; Swietach et al., 2008; McIvor et al., 2018), a smaller diffusion being possibly associated to the more tubular part. In addition, we also tested the effect of considering a higher diffusion coefficient (D_S=110 um^2/s), thought to account for the cisternae part of the ER lumen (Thul & Falcke, 2004).

Table S1.

Parameter	*Value*	*Unit*	*Description*	*Reference*
H_J	15	nm	Height ER-PM junction	Wu et al., 2006
H_S	485	nm	Height sub-PM ER	McIvor et al., 2018
r1	200	nm	Bottom radius sub-PM ER	
r2	100	nm	Top radius sub-PM ER	Samanta et al. 2015
r3	40	nm	Radius ORAI ring	Samanta et al. 2015

r4	30	nm	Distance ORAI to SERCA ring	
$C_{C,0}$	30	nM	Cytosol basal concentration	Diercks et al., 2018
$C_{S,0}$	400	µM	sub-PM ER initial concentration	Lewis, 2011
D_C	220	um^2/s	Diffusion coefficient cytosol	Hogan, 2015
D_S	10, 110	um^2/s	Diffusion coefficient sub-PM ER	Swietach et al., 2008 Thul & Falcke, 2004
F	96485	C/mol	Faraday's constant	
z	2		Valency of Ca^{2+} ions	
A_O	0.25	nm^2	Area ORAI channel	Parekh & Putney 2005
I_{ORAI}	2.1	fA	ORAI single channel current	Zweifach & Lewis, 1993
A_{IP3R}	0.41	nm^2	Area IP_3R	Thul & Falcke, 2004
I_{IP3R}	0.064	pA	IP_3R single channel current	Means et al., 2006
A_S	0.98	nm^2	Area SERCA pump	
K_F	0.27	µM	SERCA2b pump Ca^{2+} affinity	Lytton et al. 1992
K_R	1700	µM	SERCA pump reverse rate	Shannon et al. 2004
H2b	1.7		Hill coefficient SERCA2b	Lytton et al. 1992
Vmax	6.0E-23	mol/s	Maximal SERCA2b pump rate	Lytton et al. 1992
Q	2.6		Temperature coefficient	Shannon et al. 2004
τ_d	0.044	s	Duration microdomain	Estimated based on Diercks et al., 2018
T	1.3	s	Final time	
K_DS2	334	µM	Dissociation constant STIM2	Estimated based on Luik et al. 2008

Animations

All animations can be found at:

https://www2.ulb.ac.be/sciences/utc/animations/animations2.html

Animation S1

Simulated time evolution of Ca^{2+} profiles in the junction when opening 1 to 5 ORAI1 channels simultaneously under the conditions of a full ER. Left bar indicates the colour code, together

with the minimal and maximal concentration reached. ORAI1s open during 44 ms. The Ca^{2+} profiles stabilize very rapidly, a few ms after opening and closing of the channels.

<u>Animation S2a.</u>

Simulated time evolution of Ca^{2+} profiles in the junction resulting from the opening of 1 to 8 of the IP_3Rs adjacent to the junctions simultaneously, which in turn induces the opening of ORAI1 channels in the junctions as a result of local depletion of ER Ca^{2+}. Results obtained with the default value for Ca^{2+} diffusion coefficient in the ER ($D_S = 10\ \mu m^2/s$). Left bar indicates the colour code, together with the minimal and maximal concentration reached. IP_3Rs open during 44 ms. Upon depletion of local Ca^{2+} in the ER, which is quasi-instantaneous, ORAI1 channels open to an extent that depends on this local concentration, as defined by the function *f* (see equ. S13). ORAI1 opening is assumed to occur immediately after depletion because ORAI1-STIM2 aggregates are pre-formed (Diercks et al, 2018).

<u>Animation S2b.</u>

Simulated time evolution of Ca^{2+} profiles in the junction resulting from the opening of 1 to 8 of the IP_3Rs adjacent to the junctions simultaneously, which in turn induces the opening of ORAI1 channels in the junctions as a result of local depletion of ER Ca^{2+}. Results obtained with a faster Ca^{2+} diffusion coefficient in the ER ($D_S = 110\ \mu m^2/s$). Left bar indicates the colour code, together with the minimal and maximal concentration reached. IP_3Rs open during 44 ms. Upon depletion of local Ca^{2+} in the ER, which is quasi-instantaneous, ORAI1 channels open to an extent that depends on this local concentration, as defined by the function *f* (see equ. S13). ORAI1 opening is assumed to occur immediately after depletion because ORAI1-STIM2 aggregates are pre-formed (Diercks et al, 2018).

<u>Animation S3a.</u>

Simulated time evolution of the cross-sectional Ca^{2+} profiles along the z-axis resulting from the opening of 1 to 8 of the IP_3Rs. Ca^{2+} profiles in the junction, in the cytosol adjacent to the junction and in the sub-PM ER with the default value for Ca^{2+} diffusion coefficient in the ER ($D_S = 10\ \mu m^2/s$). Local depletion of ER Ca^{2+} provokes the opening of the nearby ORAI1s. This situation corresponds to the one shown in Animation S2a. The right bar indicates the colour

code of Ca^{2+} concentration in the cytosol while the left bar indicates the colour code of Ca^{2+} concentration in the ER.

Animation S3b.

Simulated time evolution of the cross-sectional Ca^{2+} profiles along the z-axis resulting from the opening of 1 to 8 of the IP_3Rs. Ca^{2+} profiles in the junction, in the cytosol adjacent to the junction and in the sub-PM ER with a larger Ca^{2+} diffusion coefficient in the ER ($D_S = 110\ \mu m^2/s$). Local depletion of ER Ca^{2+} provokes the opening of the nearby ORAI1s. This situation corresponds to the one shown in Animation S2b. The right bar indicates the colour code of Ca^{2+} concentration in the cytosol while the left bar indicates the colour code of Ca^{2+} concentration in the ER.

Animation S4a.

Simulated time evolution of the three-dimensional Ca^{2+} profiles in the whole geometry resulting from the opening of 1 to 8 of the IP_3Rs. Ca^{2+} profiles in the junction, in the cytosol adjacent to the junction and in the sub-PM ER with the default value for Ca^{2+} diffusion coefficient in the ER ($D_S = 10\ \mu m^2/s$). Local depletion of ER Ca^{2+} provokes the opening of the nearby ORAI1s. This situation corresponds to the one shown in Animation S2a and S3a. The right bar indicates the colour code of Ca^{2+} concentration in the cytosol while the left bar indicates the colour code of Ca^{2+} concentration in the ER.

Animation S4b.

Simulated time evolution of the three-dimensional Ca^{2+} profiles in the whole geometry resulting from the opening of 1 to 8 of the IP_3Rs. Ca^{2+} profiles in the junction, in the cytosol adjacent to the junction and in the sub-PM ER with a faster Ca^{2+} diffusion coefficient in the ER ($D_S = 110\ \mu m^2/s$). Local depletion of ER Ca^{2+} provokes the opening of the nearby ORAI1s. This situation corresponds to the one shown in Animation S2b and S3b. The right bar indicates the colour code of Ca^{2+} concentration in the cytosol while the left bar indicates the colour code of Ca^{2+} concentration in the ER.

Animation S5a.

Simulated time evolution of the three-dimensional Ca^{2+} profiles resulting from the opening of 1 to 8 of the IP_3Rs. The boundary wall between the junction and the rest of the cytosol is

removed for visualisation purposes. Ca^{2+} profiles in the junction, in the cytosol adjacent to the junction and in the sub-PM ER with the default value for Ca^{2+} diffusion coefficient in the ER ($D_S = 10\ \mu m^2/s$). Local depletion of ER Ca^{2+} provokes the opening of the nearby ORAI1s. This situation corresponds to the one shown in Animation S2a, S3a and S4a. The right bar indicates the colour code of Ca^{2+} concentration in the cytosol while the left bar indicates the colour code of Ca^{2+} concentration in the ER.

<u>Animation S5b.</u>

Simulated time evolution of the three-dimensional Ca^{2+} profiles resulting from the opening of 1 to 8 of the IP_3Rs. The boundary wall between the junction and the rest of the cytosol is removed for visualisation purposes. Ca^{2+} profiles in the junction, in the cytosol adjacent to the junction and in the sub-PM ER with a faster Ca^{2+} diffusion coefficient in the ER ($D_S = 110\ \mu m^2/s$). Local depletion of ER Ca^{2+} provokes the opening of the nearby ORAI1s. This situation corresponds to the one shown in Animation S2b, S3b and S4b. The right bar indicates the colour code of Ca^{2+} concentration in the cytosol while the left bar indicates the colour code of Ca^{2+} concentration in the ER.

<u>Animation S6a.</u>

Simulated time evolution of the three-dimensional Ca^{2+} profiles in the junction and Sub-PM ER resulting from the opening of 1 to 8 of the IP_3Rs. Ca^{2+} profiles in the junction, in the cytosol adjacent to the junction and in the sub-PM ER with the default value for Ca^{2+} diffusion coefficient in the ER ($D_S = 10\ \mu m^2/s$). Local depletion of ER Ca^{2+} provokes the opening of the nearby ORAI1s. This situation corresponds to the one shown in Animation S2a, S3a, S4a and S5a. The right bar indicates the colour code of Ca^{2+} concentration in the cytosol while the left bar indicates the colour code of Ca^{2+} concentration in the ER.

<u>Animation S6b.</u>

Simulated time evolution of the three-dimensional Ca^{2+} profiles in the junction and Sub-PM ER resulting from the opening of 1 to 8 of the IP_3Rs. Ca^{2+} profiles in the junction, in the cytosol adjacent to the junction and in the sub-PM ER with a faster Ca^{2+} diffusion coefficient in the ER ($D_S = 110\ \mu m^2/s$). Local depletion of ER Ca^{2+} provokes the opening of the nearby ORAI1s. This situation corresponds to the one shown in Animation S2b, S3b, S4b and S5b. The right bar

indicates the colour code of Ca^{2+} concentration in the cytosol while the left bar indicates the colour code of Ca^{2+} concentration in the ER.
